# Veterinary Register

**Published:** 1902-06

**Authors:** 


### VETERINARY REGISTER.

[See Editorial, Journal of Comparative Medicine and Veterinary Archives, July, 1901, page 438.]

[In the following list the names and data printed without comment are those furnished to us by postal card with the signature of the individual.

Those with an asterisk [*] are those to whom we have sent, by United States mail, an envelope with prepaid postage and a request for return if not delivered in ten days; as the envelopes have not been returned, we presume that the letter has been delivered, that the individual in question exists, but that he has neglected to return the enclosed postal card with the information requested.—Ed. Journal.]

### PENNSYLVANIA.

(Continued from page 336.)

| | | | | |
|---|---|---|---|---|
| Wight, W. E. | 813-15 Forbes av | Pittsburg | Ontario Vet., 1883<br>McKillip Vet., 1899 | Allegheny, 1899 |
| Wilcox, Parris | ...... | Covington | * | ...... |
| Wilkinson, J. W. | ...... | Meadville | * | ...... |
| Will, Daniel S | ...... | Bainbridge | * | ...... |
| Willard, Samuel B. | ...... | Yardley | V. D. U. Pa., 1889 | Bucks, 1889 |
| Williams, Charles | 1321 Thompson | Philadelphia | V. D. U. Pa., 1887 | Philadelphia |
| Williams, Edward | ...... | Hanover Twp. | * | ...... |
| Willtraut, F. A. | S. Wash'ton st<br>Care Nagle's Livery Stable | Wilkesbarre | * | ...... |
| Wilson, D. C. | ...... | Cassville | * | ...... |
| Wilson, Samuel | ...... | Somerfield | * | ...... |
| Wilson, Walter H. | ...... | Hatboro | N. Y. C. V. S., 1892 | Montgomery, 1893 |
| Wilson, Wm. M. | Jefferson st | Hartstown | Ontario Vet., 1898 | Crawford, 1899 |
| Winters, E. A. | ...... | Shrewsbury Twp. | * | ...... |
| Wisdom, R. | 1438 S. Broad st | Philadelphia | * | ...... |
| Wise, J. H. | ...... | Rife | * | ...... |
| Witmer, H. W. | S. Railroad st | Shippensb'g | Ontario Vet., 1895 | Cumberland, 1897 |
| Witmer, John M. | ...... | Quarryville | * | ...... |
| Wolf, John | ...... | Bethel | * | ...... |
| Wolfinger, W. W. | ...... | Ottsville | * | ...... |
| Work, Alex. S. | ...... | Marion Centre | * | ...... |
| Wright, Francis H. | ...... | Coatesville | * | ...... |
| Wright, Harry A. | 604 Ligonier st | Latrobe | * | ...... |
| Wright, W. L. | ...... | Jennerstown | * | ...... |
| Wright, W. S. | 22 N. Diamond | Allegheny | * | ...... |
| Yeager, Conrad | ...... | St. Bonifacius | * | ...... |
| Yeagle, Jos. S. | ...... | Warrensville | * | ...... |
| Yeakle, Emanuel | ...... | Sylvan | * | ...... |
| Yetter, Ed. G. | ...... | Toughken-amon | * | ...... |
| Yingst, Henry | ...... | Bellgrove | * | ...... |
| Yingst, Walter H. | 2d & Walnut | Harrisburg | N. Y. C. V. S., 1896 | Dauphin |
| Yocum John F. | ...... | Alexandria | * | ...... |
| Yoder, Edwin C. | ...... | Athol | * | ...... |
| Yoder, Henry S. | ...... | Manatawny | * | ...... |
| Yohn, Thos. M. | ...... | Spruce Hill | * | ...... |
| Yost, Augustine, Sr. | ...... | Carrolltown | * | ...... |
| Yost, Jacob R. | ...... | Manchester | * | ...... |
| Young, Wm., Jr. | ...... | Nanticoke | * | ...... |
| Young, E. | 236 E. 4th st | Chester | * | ...... |
| Young, Geo. J. | Chestnut st | Coatesville | Ontario Vet., 1893 | Chester, 1893 |
| Young, Thos. D. | ...... | Media | * | ...... |
| Young, T. D. & Bro. | 236 E. 4th st | Chester | * | ...... |
| Young, W. W. | ...... | Philadelphia | * | ...... |
| Young, Wm., Sr. | ...... | Nanticoke | * | ...... |
| Zell, Daniel | ...... | Churchtown | * | ...... |
| Zellers, I. W. | 404½ Broad st | Harrisburg | Dauphin, 1889 | ...... |

